# Rarity within rarity: complete response to chemoimmunotherapy in a patient with polymerase epsilon (POLE)-mutated locally advanced jejunal carcinoma

**DOI:** 10.1093/oncolo/oyag223

**Published:** 2026-06-04

**Authors:** Michele Prisciandaro, Federica Morano, Davide Citterio, Maria Flores Reyes, Marco Barella, Vincenzo Mazzaferro, Filippo Pietrantonio

**Affiliations:** Medical Oncology Department, Fondazione IRCCS Istituto Nazionale dei Tumori, Milan, 20133, Italy; Medical Oncology Department, Fondazione IRCCS Istituto Nazionale dei Tumori, Milan, 20133, Italy; Hepatobiliary Pancreatic, Upper GI and Liver Transplantation Unit, Fondazione IRCCS Istituto Nazionale dei Tumori, Milan, 20133, Italy; Hepatobiliary Pancreatic, Upper GI and Liver Transplantation Unit, Fondazione IRCCS Istituto Nazionale dei Tumori, Milan, 20133, Italy; Department of Diagnostic Innovation, Pathology Unit 1, Fondazione IRCCS Istituto Nazionale dei Tumori, Milan, 20133, Italy; Hepatobiliary Pancreatic, Upper GI and Liver Transplantation Unit, Fondazione IRCCS Istituto Nazionale dei Tumori, Milan, 20133, Italy; Medical Oncology Department, Fondazione IRCCS Istituto Nazionale dei Tumori, Milan, 20133, Italy

**Keywords:** immune checkpoint inhibitor, tumor mutation burden, gastrointestinal cancer, polymerase epsilon

## Abstract

**Background:**

Jejunal carcinoma is an exceptionally rare malignancy with limited therapeutic options beyond chemotherapy. Polymerase epsilon (*POLE*) mutations in this tumor type are even rarer, yet they may represent a predictive biomarker for response to immune checkpoint inhibitors in jejunal cancer and agnostically.

**Case presentation:**

We report a 58-year-old male patient diagnosed with a POLE-mutated jejunal carcinoma. He presented with left-sided abdominal pain and was found to have an extremely large mass in the left upper quadrant. Biopsy confirmed jejunal adenocarcinoma. He was initially treated with two cycles of FOLFOX, and upon detection of the POLE mutation, nivolumab was added. After four cycles of chemo-immunotherapy, imaging demonstrated significant tumor regression. However, radiologic and clinical signs of subocclusion prompted treatment discontinuation and surgical resection. Histopathologic analysis revealed a complete pathological response, with no viable tumor cells. The patient has been disease-free since January 2025.

**Conclusion:**

This case underscores the role of immunotherapy in *POLE*-mutated tumors regardless of the site of origin and highlights the potential usefulness of molecular profiling in rare malignancies. In line with the agnostic approach used for microsatellite instability, molecular-driven clinical trials should be prioritized over histology-based studies to optimize treatment strategies for these orphan diseases.

Key points

- POLE exonuclease domain mutations define an ultra-hypermutated phenotype associated with high sensitivity to immune checkpoint inhibitors.
- Comprehensive genomic profiling can uncover actionable alterations even in rare malignancies lacking standard therapeutic options.
- This case highlights a complete pathological response to chemo-immunotherapy in a POLE-mutated jejunal carcinoma, an extremely rare entity.
- Rapid tumor regression during immunotherapy may lead to clinically relevant complications, such as bowel subocclusion in anatomically predisposed lesions.

## Introduction

Jejunal carcinoma is an exceptionally rare malignancy, representing a small subset of small bowel cancers, which in total account for less than 5% of all gastrointestinal tumors.^1^ Due to its rarity and nonspecific symptoms, it is often diagnosed at an advanced stage, as early symptoms are nonspecific and can mimic benign conditions such as irritable bowel syndrome or peptic ulcer disease. In localized disease, surgery remains the standard of care, while chemotherapy is the mainstay for advanced cases, given the paucity of clinical trials or case series evaluating targeted or immunotherapeutic approaches in small bowel cancers.^2^

POLE mutations occurring in the exonuclease domain are associated with an ultra-mutated phenotype and exceptionally high tumor mutational burden (TMB),^3^ leading to increased neoantigen presentation and enhanced immunogenicity. This ultra-mutated state shares some similarities with microsatellite instability (MSI),^4^ which is validated as a predictive biomarker for immune checkpoint inhibitors (ICIs).^5^ MSI-high tumors exhibit defective DNA mismatch repair and (dMMR), *POLE*-mutant tumors have deficient proofreading activity, leading to heightened immune response and susceptibility to Programmed Death-1 (PD-1)/Programmed Death Ligand-1 (PD-L1) blockade.^6^ Although the role of ICIs in *POLE*-mutated small intestine carcinomas cannot be specifically investigated due to extreme rarity, there is a strong rationale for considering *POLE* mutations as an agnostic predictive biomarker for immunotherapy response.

Here, we describe a case of a *POLE*-mutated unresectable and locally advanced jejunal carcinoma achieving complete pathological response following chemo-immunotherapy, suggesting a potential role for ICIs in this rare subset as an agnostic approach.

## Patient history

A 58-year-old male presented in August 2024 with left-sided abdominal pain and a palpable mass following physical exertion. Initial imaging, including ultrasound and contrast-enhanced computed tomography (CT), revealed a large mass in the left upper quadrant, suggestive of a jejunal origin. Subsequent magnetic resonance imaging confirmed the presence of satellite nodules, and the disease was judged as unresectable. Percutaneous biopsy of the lesion identified a poorly differentiated carcinoma with an immunophenotype consistent with small bowel origin, showing positivity for CKAE1/AE3, CK20, and CDX2 and negativity for CK7, neuroendocrine, hematolymphoid, melanocytic, germ-cell, and mesenchymal markers, confirming the jejunal origin. No elevation of serum tumor markers, including CEA and CA 19-9, was observed at diagnosis. The patient started systemic therapy with FOLFOX for two cycles, with initial symptomatic benefit.

## Molecular tumor board

Comprehensive genomic profiling was performed using next-generation sequencing with the Oncomine Comprehensive Assay Plus (OCA Plus; Thermo Fisher Scientific), a targeted panel designed to detect single nucleotide variants, insertions/deletions, copy number alterations, and gene fusions and to estimate TMB. Molecular analysis revealed an MSS tumor, without detectable alterations in HER2 or BRAF. A KRAS mutation (A59T) was identified. Notably, a pathogenic POLE exonuclease domain mutation (S459Y) was detected, associated with an ultra-hypermutated phenotype, with a tumor mutational burden of 238 mutations/Mb. These findings were discussed in a multidisciplinary molecular tumor board. Despite the absence of microsatellite instability, the presence of a POLE exonuclease domain mutation and the extremely elevated TMB were considered indicative of a highly immunogenic tumor phenotype, potentially sensitive to immune checkpoint inhibition.

## Patient update

**Figure 1.**
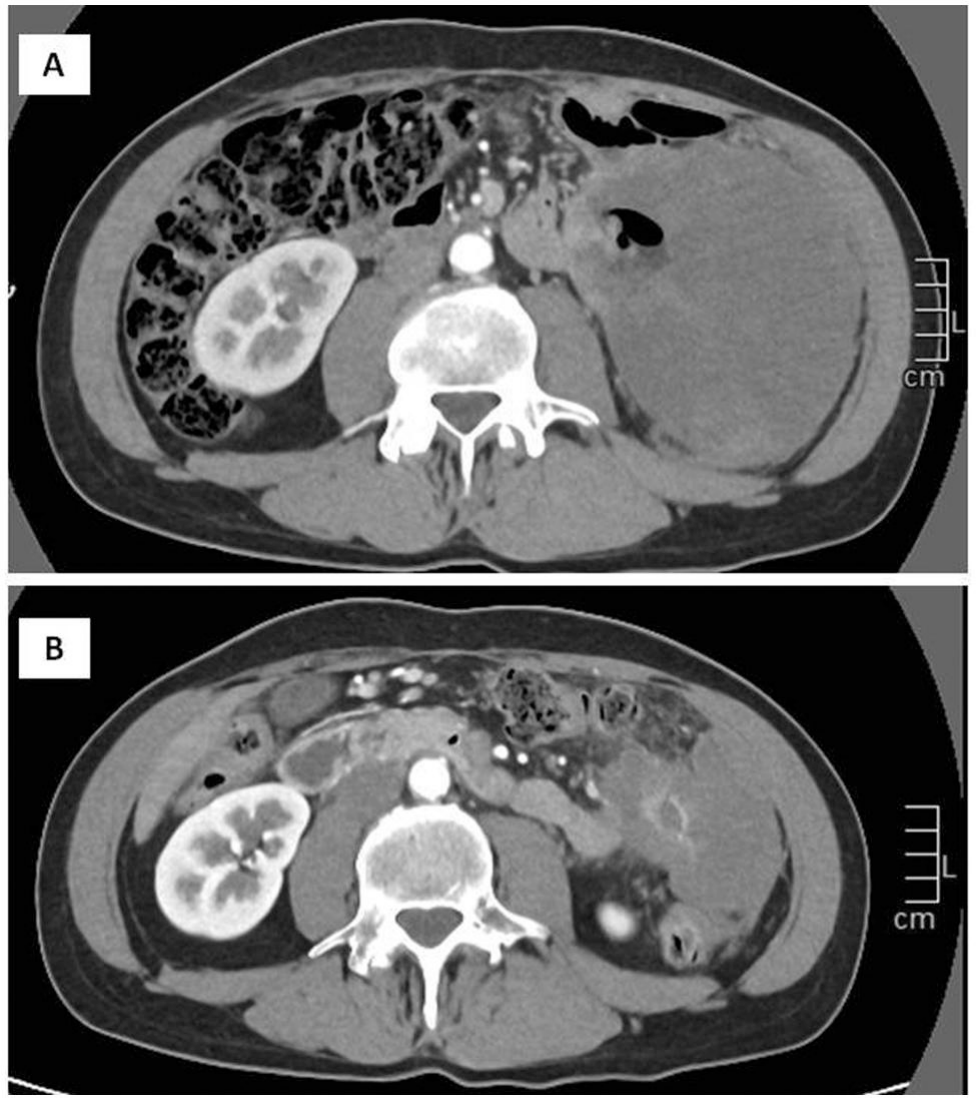
CT scan of jejunal lesion at baseline (A) and after (B) chemo-immunotherapy.

**Figure 2.**
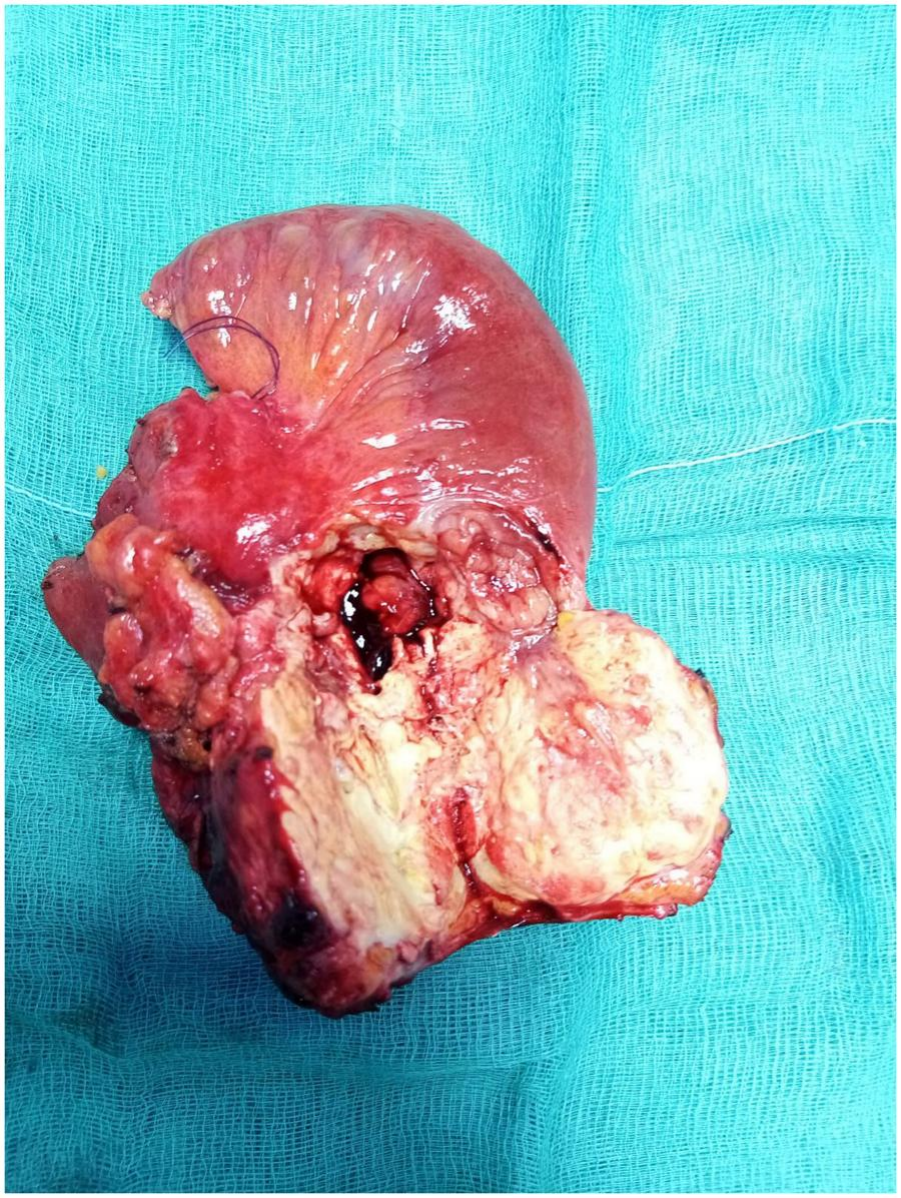
Macroscopic histopathologic examination.

**Figure 3.**
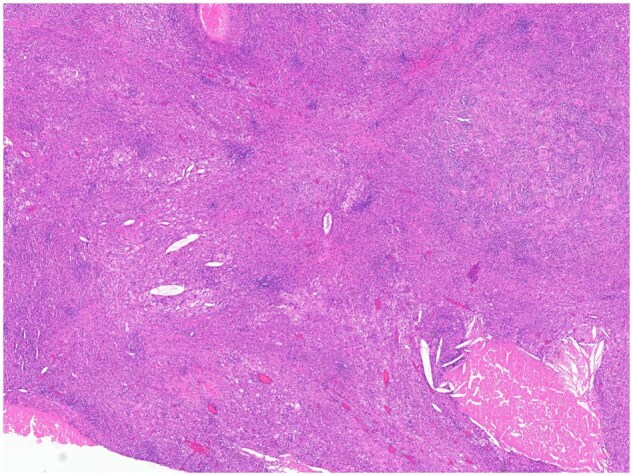
Microscopic histological examination: small intestine wall full thickness site of ulceration, necrosis, fibrosis, lympho-plasmocytic and histiocytic inflammatory infiltrate (20×). No evidence of residual neoplastic cell (complete pathological response).

**Figure 4.**
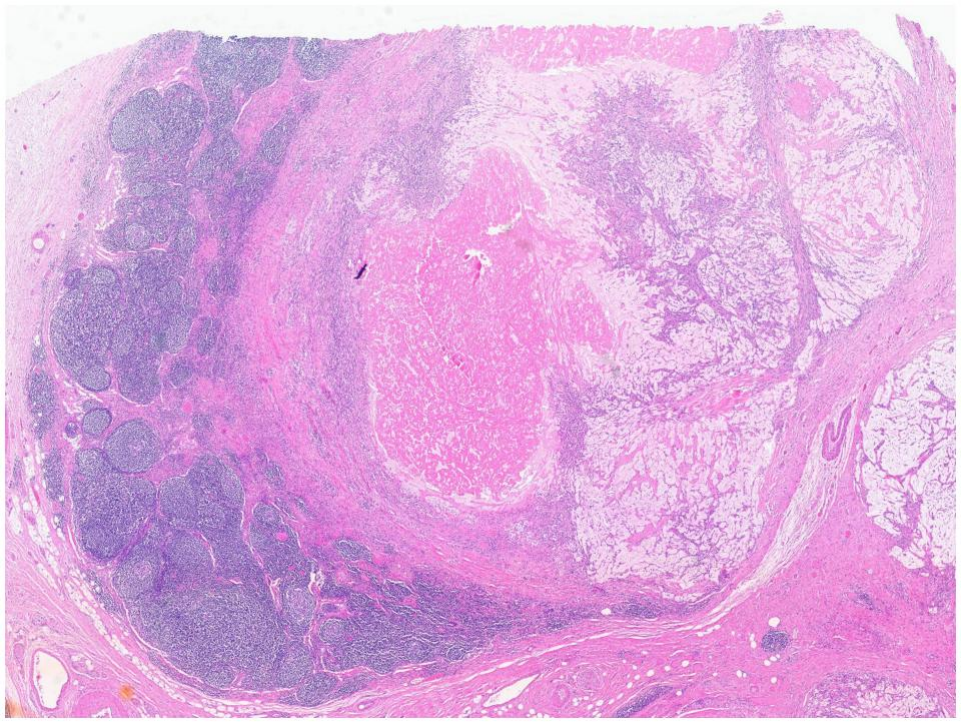
Mesenteric lymph node in post-treatment outcomes, site of necrosis, “mucin lakes” and lympho-plasmocytic inflammatory infiltrate (20×). No evidence of residual neoplastic cell (complete pathological response).

In the absence of established targeted therapeutic options for advanced jejunal carcinoma, a biomarker-driven, histology-agnostic approach was adopted, and the addition of the anti–PD-1 agent nivolumab to chemotherapy was recommended. Shortly after treatment initiation, the patient experienced a clear clinical benefit, with progressive reduction of the palpable abdominal mass until it became no longer detectable on physical examination. However, one week after the second chemo-immunotherapy cycle the patient developed recurrent postprandial vomiting, prompting hospital admission and further evaluation. A restaging CT scan (Figure 1) was then performed, demonstrated a dramatic tumor response (50% shrinkage according to Response Evaluation Criteria in Solid Tumors); nonetheless, at the same time, imaging revealed signs of subocclusion of the involved intestinal segment. Subsequently, fluoroscopic imaging confirmed jejunal transit delay due to substenosis. According to this, to prevent complete obstruction and potential risk of perforation, the treatment was stopped. In a few days a surgical reevaluation was then performed, considering also the major radiologic response observed, with the indication for radical surgery. So, after four weeks from the last chemo-immunotherapy administration, in January 2025, en bloc resection of the affected jejunal loop with mesenteric lymphadenectomy was performed. At the macroscopic examination of the resected specimen, an extensively necrotic tumor mass with “cheesy” consistency was observed (Figure 2). Histopathological examination demonstrated a pathological complete response, with absence of viable tumor cells associated with ulceration, fibrosis, and necrosis extending through the full thickness of the intestinal wall. A dense inflammatory infiltrate of lymphocytes, plasma cells, histiocytes, and neutrophils was present. Two perivisceral lymph nodes exhibited fibrosis, histiocytic reaction, and focal necrosis, but no residual malignancy (Figures 3 and 4). Given the complete response, close clinical follow-up was recommended, including clinical evaluation, serum tumor marker assessment, and CT imaging every 3 months. Since February 2025, the patient has remained disease-free with no evidence of recurrence.

## Discussion

Jejunal carcinoma is a rare malignancy, and therapeutic options for advanced-stage disease are largely restricted to systemic chemotherapy.^2^ However, complete and durable responses and thus potential curative outcomes remain uncommon.^7^ The occurrence of *POLE* mutations in jejunal carcinoma appears to be exceptionally rare and, to our knowledge, has not been previously reported. However, it may represent an agnostic predictive biomarker for response to immunotherapy. *POLE* encodes the catalytic subunit of DNA polymerase epsilon, responsible for high-fidelity DNA replication and repair.^3^^,^^8^ Mutations in the exonuclease domain lead to an ultra-mutated phenotype with extremely high TMB, usually superior to 100 Mut/Mb, and increased neoantigen presentation and tumor immunogenicity.^9^ In our case, the tumor exhibited an extremely high TMB of 238 mut/Mb, driven by the presence of a *POLE* mutation, defining an highly ultramutated phenotype. Consequently, *POLE*-mutant tumors are highly responsive to ICIs, such as nivolumab, which reinvigorate T-cell-mediated anti-tumor responses. Studies have shown that *POLE-*mutated tumors exhibit increased CD8+ tumor-infiltrating lymphocytes and upregulated immune checkpoint pathways, reinforcing their sensitivity to ICIs.^6^^,^^10^ Additionally, *POLE* mutations have been associated with improved response to PD-1/PD-L1 blockade across multiple cancer types, including colorectal and endometrial cancers.^11–14^ The remarkable response in this case suggests that the presence of POLE mutations may be an optimal subset for immunotherapy even in jejunal carcinomas. This case also underscores the broader potential role of immunotherapy in POLE-mutant orphan tumors currently lacking effective treatments. This is why current NCCN guidelines include immune checkpoint inhibitors among treatment options for advanced or unresectable small bowel adenocarcinoma harboring dMMR/MSI-H or POLE/POLD1 alterations, although evidence remains limited and largely extrapolated from colorectal cancer. From an agnostic perspective, as seen with MSI-high and NTRK fusion-positive tumors,^15^^,^^16^ molecular profiling should be increasingly prioritized. Future clinical trials should be designed based on molecular alterations rather than histology alone, allowing for the identification of patients most likely to benefit from targeted and immune-based therapies, particularly for rare neoplasms like jejunal cancer. While the response observed represents a clear therapeutic success, it also reminds us that profound tumor responses may carry clinically relevant consequences. Indeed, this case highlights the potential risks related to a highly effective immunotherapy, particularly in anatomically narrow or stricturing lesions. Bowel obstruction during immunotherapy response has recently been described in patients with dMMR colon cancer treated with immune checkpoint inhibitors, where luminal obstruction occurred paradoxically in the context of marked tumor regression. In tumors with preexisting circumferential involvement or radiologic luminal narrowing, rapid immune-mediated tumor necrosis may induce marked inflammatory edema followed by fibrotic remodeling, leading to paradoxical luminal compromise despite radiological tumor regression.^17^ Although our case involved a jejunal primary tumor, a similar mechanism is plausible, as significant radiologic response was accompanied by progressive luminal compromise requiring surgery. Careful clinical and radiologic monitoring should therefore be considered in patients with stricturing small bowel lesions undergoing immunotherapy. So, in conclusion, this case represents a rare instance of a complete pathological response in a *POLE*-mutated jejunal carcinoma treated with short-course FOLFOX and nivolumab. These findings highlight the need for expanded molecular profiling in rare malignancies and support the rationale for designing agnostic, biomarker-driven clinical trials to optimize treatment strategies. At the same time, profound immune-related tumor response may entail clinically relevant complications, underscoring the need for careful multidisciplinary monitoring.

## Data Availability

The data underlying this article will be shared on reasonable request to the corresponding author.
